# A Rare and Challenging Presentation of Acute Hemorrhagic Leukoencephalitis With Tumefactive Demyelinating Lesions in a 41-Year-Old Male

**DOI:** 10.7759/cureus.58282

**Published:** 2024-04-15

**Authors:** Samar Iltaf Mairajuddin, Jihad Said Salim Inshasi, Raheel Muneer Ahmed Channa, Shaista Anwar Siddiqi, Abubaker Abdul Rahman Shaffi Al Madani, Raya Flayyih

**Affiliations:** 1 Neurology, Rashid Hospital, Dubai, ARE; 2 Research, Dubai Medical College for Girls, Dubai, ARE

**Keywords:** susceptibility weighted imaging (swi), apparent diffusion coefficient (adc), diffusion-weighted imaging (dwi), acute hemorrhagic leukoencephalitis ahle, tumefactive ms, corticosteroid therapy, magnetic resonance imaging, cns demyelinating disease

## Abstract

Acute hemorrhagic leukoencephalitis (AHLE) is a rare and severe inflammatory condition of the central nervous system (CNS), characterized by hemorrhagic lesions in the brain's white matter. Here, we present a case of AHLE with concurrent tumefactive demyelinating disease, highlighting the diagnostic and management challenges associated with this complex presentation. Tumefactive multiple sclerosis (MS) is a rare variant of MS characterized by large, space-occupying lesions in the CNS. Concurrently, hemorrhagic leukoencephalitis (HLE) represents a severe inflammatory disorder characterized by hemorrhagic lesions within the CNS white matter.

The diagnosis of tumefactive MS with associated HLE posed significant diagnostic challenges due to overlapping clinical and radiological features. Management involved high-dose corticosteroid therapy and supportive care measures, with longitudinal follow-up to assess treatment response and prevent complications. The patient exhibited a favorable clinical response to treatment, with gradual improvement in symptoms and resolution of radiological abnormalities. The coexistence of tumefactive MS with HLE is exceptionally rare and presents diagnostic and therapeutic challenges.

We report a 41-year-old male presenting with acute neurological symptoms, including severe headache, confusion, left-sided body weakness, slurred speech, and blurred vision. Neurological examination revealed dysarthric speech, right homonymous hemianopia, left upper motor neuron facial palsy, and motor deficits. MRI demonstrated multifocal areas of T2 hyperintensity with associated hemorrhage, suggestive of tumefactive MS with associated HLE. Diagnostic workup included neurological examination, MRI imaging, cerebrospinal fluid analysis, and serological testing. Management involved high-dose corticosteroid therapy and supportive care measures. The patient exhibited a favorable clinical response to treatment, with gradual improvement in symptoms and resolution of radiological abnormalities. Longitudinal follow-up confirmed sustained improvement.

In conclusion, the coexistence of tumefactive MS with HLE poses diagnostic challenges due to overlapping features. This case underscores the importance of considering rare and atypical presentations of CNS demyelinating disease and the potential complications, including associated HLE. Comprehensive evaluation, multidisciplinary collaboration, and individualized management are essential for optimizing outcomes in patients with complex CNS inflammatory disorders.

## Introduction

Tumefactive demyelinating lesions (TDLs) represent a rare but significant manifestation of central nervous system (CNS) demyelination, characterized by the presence of large (>2 cm), tumor-like lesions with perilesional edema, mass effect, and/or broken ring enhancement on MRI imaging. The prevalence of TDLs within the multiple sclerosis (MS) population has been reported to be approximately 1-2 per 1000 cases, indicating their relatively rare occurrence [1]. These lesions often present diagnostic challenges, necessitating a careful differential diagnosis to rule out other space-occupying lesions, including primary and metastatic tumors, abscesses, and vascular malformations [2].

Hemorrhagic leukoencephalitis (HLE) is a rare and potentially fatal inflammatory disorder of the CNS, characterized by multifocal hemorrhagic lesions within the white matter. Demyelinating diseases, such as MS and acute disseminated encephalomyelitis (ADEM), represent another group of immune-mediated disorders affecting the myelin sheaths in the CNS. While traditionally considered distinct entities, there is increasing recognition of cases exhibiting features of both HLE and demyelinating disease, leading to diagnostic and therapeutic challenges [3].

In this report, we discuss the occurrence of hemorrhage in a patient with tumefactive MS. Neovascularization may contribute to tissue repair, particularly in large inflammatory cerebral lesions with increased vascular fragility [4].

In recent years, several case reports and studies have shed light on the association between HLE and demyelinating disease, providing insights into clinical presentation, radiological findings, pathophysiological mechanisms, and management strategies. For instance, a case report described a patient with concomitant HLE and ADEM, highlighting the overlapping clinical and radiological features observed in these conditions [5]. Similarly, a study reported cases of HLE with features suggestive of MS, underscoring the diagnostic ambiguity and need for comprehensive evaluation in such cases [6].

The pathophysiological mechanisms underlying the association between HLE and demyelinating disease remain poorly understood. Dysregulated immune responses, including cytokine release and complement activation, are hypothesized to play a role in blood-brain barrier disruption, inflammation, and subsequent hemorrhagic injury. In the context of demyelinating disease, aberrant immune responses targeting myelin sheaths may predispose individuals to blood-brain barrier dysfunction and hemorrhagic complications [7].

The underlying pathophysiological mechanisms and optimal management strategies for this unique presentation remain poorly understood. This case report aims to present a rare case of CNS demyelinating disease with acute hemorrhagic leukoencephalitis (AHLE) and discuss the challenges associated with diagnosis and management.

## Case presentation

A 41-year-old gentleman with no prior history of neurological disorders presented to the emergency room with acute onset severe symptoms. He reported experiencing a week of fever and sore throat preceding the onset of his current symptoms. There was no history of recent trauma or significant medical illnesses. The patient complained of a severe headache, confusion, left-sided body weakness, slurred speech, and blurred vision. These symptoms emerged abruptly just two hours before he sought medical attention, prompting concern for a neurological emergency. On neurological examination, the patient displayed severe dysarthric speech, he has gaze preference to the right and exhibited right homonymous hemianopia, indicative of visual impairment. Right eye visual acuity reduced to 20/200. Ophthalmoscopic examination of the disc was normal. He also presented with left upper motor neuron-type facial palsy. Motor examination revealed reduced power in the left upper and lower limbs, graded as 2/5 in the upper limb and 3/5 in the lower limb. Additionally, left-sided hemi-sensory neglect was observed, along with brisk deep tendon reflexes and an extensor left plantar reflex. The patient's National Institutes of Health Stroke Scale (NIHSS) score was recorded as 16.

Laboratory investigations revealed elevated erythrocyte sedimentation rate (ESR) and C-reactive protein (CRP). Cerebrospinal fluid (CSF) analysis showed elevated protein levels and the presence of oligoclonal bands (OCBs), while autoimmune panels and infectious disease markers were negative. Notably, delayed visual evoked potentials and prolonged P100/P1 latency were observed on the right side.

**Figure 1:**
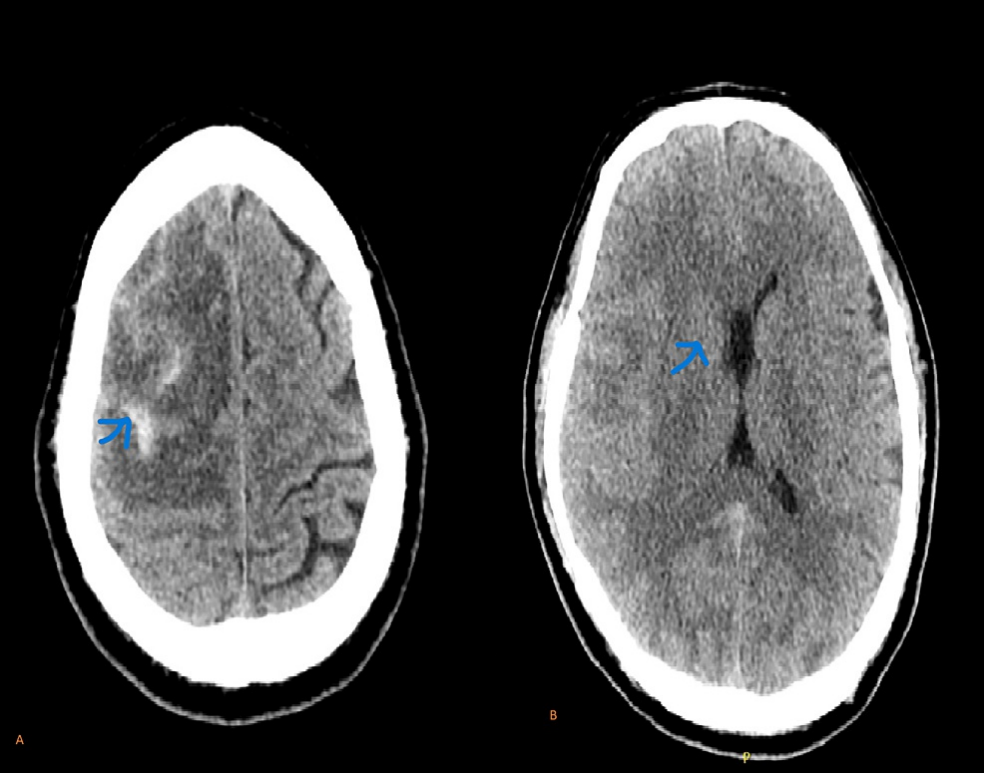
CT scan of the brain in the axial view (A, B) A: Acute right frontal subarachnoid hemorrhage with perifocal edema (arrow) B: Mass effect on the frontal horn of right lateral ventricle (arrow)

Imaging studies including a CT scan brain suggest an acute right subarachnoid hemorrhage with perifocal edema with some mass effect compressing the frontal horn of the lateral ventricle (Figure 1).

**Figure 2:**
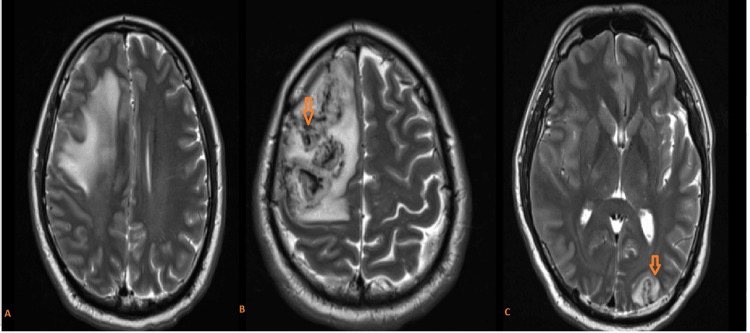
MRI brain axial T2-weighted (T2W) imaging (A, B, C) A, B: T2W imaging suggestive of large heterogeneously hyperintense signal intensity at the right frontal lobe (arrow) C: Mixed-intensity lesion is also noted at the left occipital region (arrow)

MRI brain reveals a large frontal cortical and subcortical hemorrhagic lesion with perilesional edema (Video 1). T1-weighted image (T1W) shows a hypointense lesion in the right frontal region with surrounding edema and mass effect, that is hyperintense to mixed intensity on T2-weighted (T2W) imaging (Figure 2).

**Video 1:** MRI brain T1 and T2 weighted images MRI brain axial images show a large acute hemorrhagic lesion in the right frontal lobe. Axial T1-weighted image (T1W) shows isointense to hypointense lesion in the right frontal region that is mixed intensity heterogenous on T2-weighted (T2W) imaging with perilesional edema, causing a mass effect in the form of sulcal and right lateral ventricular effacement and midline shift of 5mm to the left side. Another similar lesion is noted at the left occipital region.

**Figure 3:**
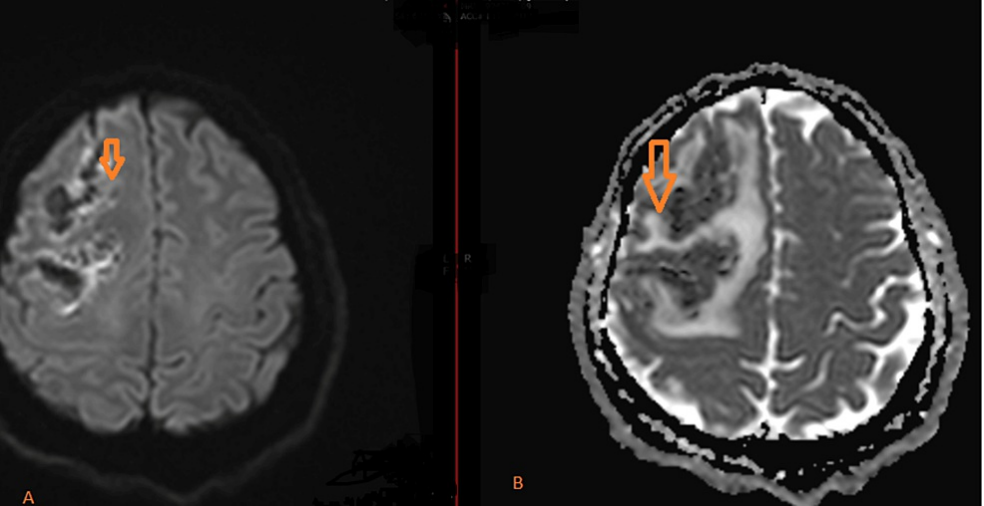
(A) Diffusion-weighted image (DWI) and (B) apparent diffusion coefficient (ADC) map (A) DWI: diffusion restriction at the peripheral edges of the lesion, confirmed by low attenuated signals on the ADC map (B) (arrow).

**Figure 4:**
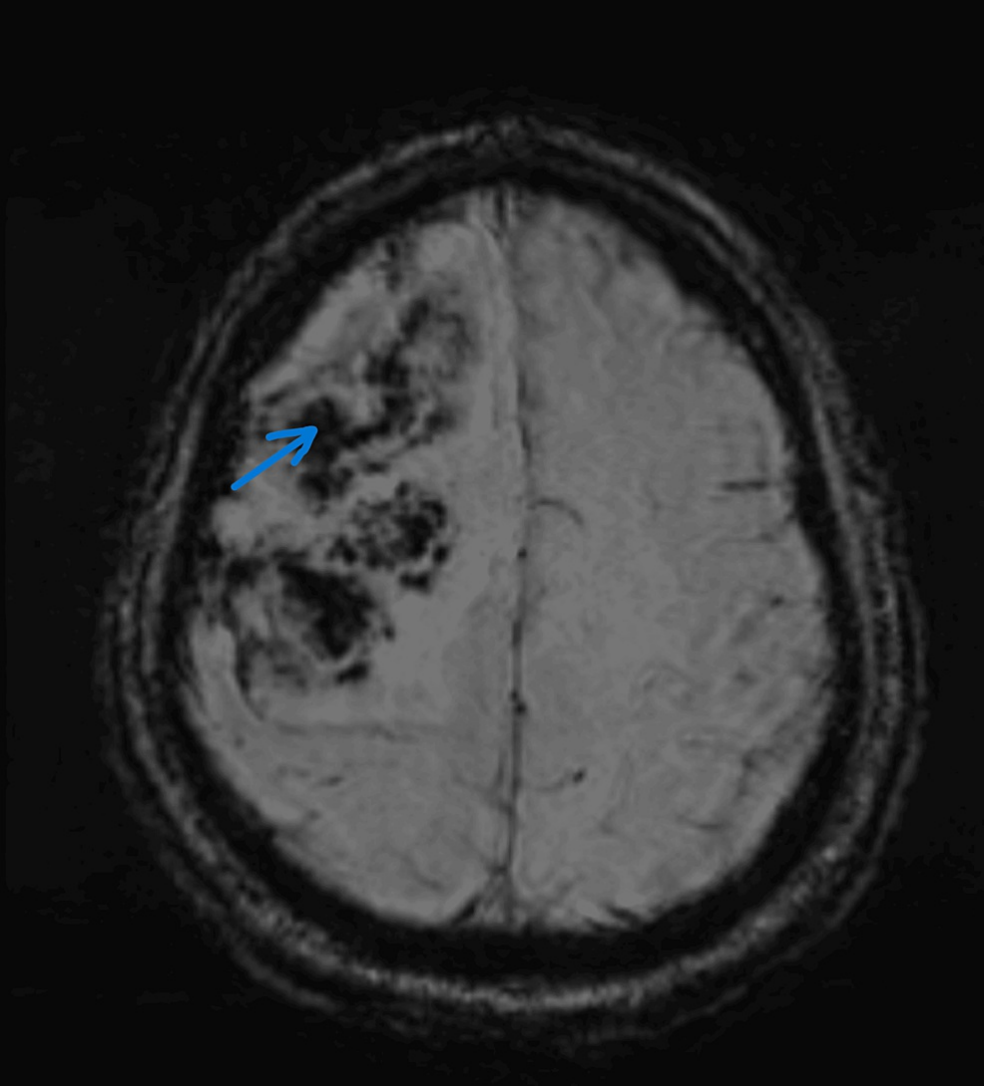
Susceptibility-weighted image (SWI) Blooming artifact (low-signal intensity) appearing on SWI images due to blood products at the right frontal lobe (arrow).

**Figure 5:**
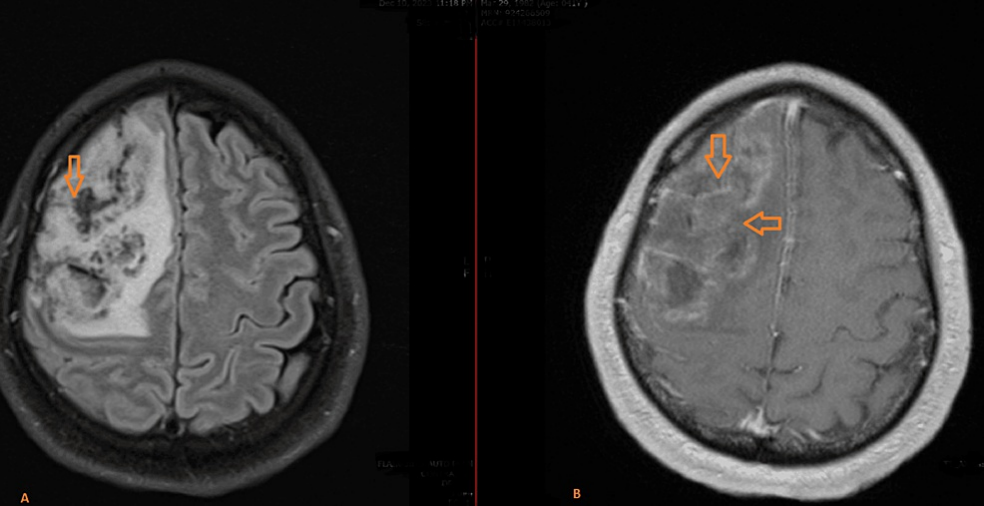
(A) Fluid-attenuated inversion recovery (FLAIR) and T1-weighted post-contrast images (B) (A) Heterogeneously hyperintense on T2-weighted and FLAIR images (arrow). (B) Axial T1-contrast image reveals a large right frontal lobe lesion with an incomplete ring of enhancement; the incomplete ring opens on the gray matter side of the lesion (arrow).

Diffusion restriction noted at the peripheral edges of the lesion, compared to low attenuated signals on the apparent diffusion coefficient (ADC) map (Figure 3). Blooming artifacts appeared as low signal intensity due to blood products on susceptibility-weighted imaging (SWI) images (Figure 4). Subsequent MRI of the brain post-contrast demonstrated multiple large lesions, with incomplete ring enhancement, with the pattern of the incomplete rings oriented towards the gray matter (Figure 5).

A normal peak of N-acetyl aspartate (NAA) and an increased lactate-to-choline peak were detected by magnetic resonance spectroscopy (MRS). An increased choline (CHO)/NAA ratio with an increased lactate peak supports the diagnosis of demyelinating disease. However, CT angiography/venography is not suggestive of cerebral thrombosis, and no arteriovenous (AV) malformation or aneurysm was detected with a normal circle of Willis. CT scan of the abdomen, pelvis, and chest was unremarkable.

The patient was promptly started on high-dose intravenous methylprednisolone therapy (1000 mg × 5/day) to mitigate the inflammatory response and reduce neurological symptoms. Additionally, supportive care measures were initiated to address any associated complications, such as pain management and monitoring for signs of increased intracranial pressure.

The patient demonstrated a positive response to corticosteroid therapy, with gradual improvement in symptoms throughout hospitalization. Oral prednisolone was continued and tapered off over several months to prevent disease relapse. Follow-up neurological assessments and imaging studies indicated resolution of the acute inflammatory process, with no evidence of new lesions or disease progression.

The brain biopsy was recommended, but the patient refused the procedure. However, the patient's functional status improved significantly, and he was discharged with an improved Expanded Disability Status Scale (EDSS) score.

Follow-up MRI brain after 15 days shows significant near-to-complete regression of the mass effect and resolution of the signal intensity related to blood products in the right frontal lesion (Video 2).

**Video 2:** Follow-up of the MRI brain (15 days after starting treatment) Compared to the prior MRI, there is a significant near-complete regression of the mass effect upon the third and right lateral ventricles. There has been a significant partial regression of the right frontal gyral swelling and sulcal effacement as well as regression of the signal intensity related to blood products.

## Discussion

Fulminant tumor-like demyelinating disease (FTDD) represents a rare and aggressive variant of CNS demyelination characterized by the rapid development of large, space-occupying lesions with mass effect and perilesional edema. The pathophysiology of FTDD involves complex immune-mediated processes, potential hemorrhagic transformation, and rare associations with encephalitis, necessitating a comprehensive understanding for accurate diagnosis and management.

The pathogenesis of tumefactive disease is thought to involve dysregulated immune responses targeting myelin components within the CNS [8]. Activation of pro-inflammatory cytokines and immune cells leads to demyelination, blood-brain barrier disruption, and recruitment of inflammatory cells into the CNS parenchyma. This inflammatory cascade results in tissue injury, edema formation, and mass effect, mimicking features of neoplastic tumors. The exact triggers for the rapid and fulminant course of FTDD remain unclear but likely involve a combination of genetic predisposition, environmental factors, and immunological dysregulation.

Despite their rarity, TDLs carry significant implications for patient management and prognosis. Prompt recognition and differentiation from other CNS pathologies are crucial to guide appropriate treatment strategies. High-dose corticosteroids remain the mainstay of acute management, although disease-modifying therapies may be indicated in patients with an underlying diagnosis of MS.

This introduction sets the stage for the subsequent discussion of a challenging case involving hemorrhagic tumefactive MS, highlighting the complexities associated with diagnosis and management in this unique clinical scenario.

Management strategies for patients with concurrent HLE and demyelinating disease typically involve immunomodulatory therapies aimed at suppressing inflammatory responses and preventing disease progression. High-dose corticosteroids, plasma exchange, and immunosuppressive agents may be considered in severe or refractory cases. However, optimal treatment approaches remain uncertain, emphasizing the need for individualized management and further research in this area [9].

The underlying pathophysiological mechanisms and optimal management strategies for this unique presentation remain poorly understood. This case report aims to present a rare case of tumefactive MS with AHLE and discuss the challenges associated with diagnosis and management. While hemorrhagic transformation within demyelinating lesions is rare, it can occur in the setting of FTDD, further complicating the clinical presentation and radiological interpretation [10].

Hemorrhage within demyelinating lesions may result from vascular fragility, neovascularization, or microvascular injury secondary to inflammatory processes. Additionally, FTDD may rarely be associated with concurrent encephalitis, leading to overlapping clinical features and diagnostic challenges. The coexistence of inflammatory demyelination and encephalitis underscores the heterogeneity of fulminant demyelinating diseases and highlights the importance of thorough diagnostic evaluation. Imaging plays a crucial role in the diagnosis and characterization of FTDD [11].

MRI remains the primary modality for visualizing lesion morphology, enhancement patterns, and associated edema. Tumefactive lesions typically exhibit large size (>2 cm), irregular borders, and heterogeneous enhancement on contrast-enhanced MRI. Gradient echo sequences may reveal susceptibility artifacts indicative of hemorrhage within the lesions. Advanced MRI techniques, such as diffusion-weighted imaging and perfusion imaging, may provide additional insights into lesion characteristics and vascularity.

In cases where the diagnosis of FTDD remains uncertain or when atypical features are present, a biopsy of the lesion may be considered to confirm the underlying pathology [12].

Histopathological examination of biopsy specimens typically reveals features consistent with demyelination, including perivascular lymphocytic infiltrates, myelin loss, and gliosis. Evidence of hemorrhage, such as hemosiderin deposition and extravasated erythrocytes, may also be observed. However, brain biopsy is an invasive procedure associated with inherent risks, underscoring the importance of careful patient selection and consideration of alternative diagnostic modalities [13].

The management requires a multidisciplinary approach and often involves high-dose corticosteroids as the first-line therapy to mitigate inflammation and edema. In refractory cases, plasma exchange, intravenous immunoglobulins, or immunosuppressive agents may be considered to modulate the immune response. The role of surgical intervention, such as lesion biopsy or debulking, remains controversial and should be carefully weighed against potential risks.

The prognosis of tumefactive disease with HLE varies widely and depends on factors such as lesion size, location, and response to treatment. While some patients experience partial or complete remission with immunomodulatory therapy, others may progress to irreversible neurological deficits or require long-term disability management.

## Conclusions

In conclusion, this case report highlights the diagnostic and therapeutic challenges posed by tumefactive MS with hemorrhagic conversion. Through a multidisciplinary approach involving neurology, neuroradiology, and neurosurgery, the patient received timely treatment with high-dose corticosteroids, resulting in significant clinical improvement. Close monitoring of neurological status and serial imaging studies confirmed the resolution of the hemorrhagic lesion and regression of surrounding edema over time. This case underscores the importance of considering fulminant tumefactive MS in the differential diagnosis of patients presenting with acute neurological symptoms and large, enhancing brain lesions, particularly in the context of stroke or tumor-like presentation. Early recognition and prompt initiation of appropriate treatment are crucial for optimizing outcomes and minimizing long-term disability in these patients. Further research is warranted to elucidate the underlying pathophysiological mechanisms driving hemorrhagic conversion in CNS demyelinating disease and to explore novel therapeutic strategies aimed at modulating the immune response and preventing disease progression.
